# Rare Manifestation of Idiopathic Tracheobronchopathia Osteochondroplastica: Misdiagnosed and Untreated Entity?

**DOI:** 10.7759/cureus.9407

**Published:** 2020-07-26

**Authors:** Abdul Rana, Haitem Mezughi, Shuja A Malik, Kanaan Mansoor, Amro Al-Astal

**Affiliations:** 1 Internal Medicine, Marshall University Joan C. Edwards School of Medicine, Huntington, USA; 2 Pulmonology, Marshall University Joan C. Edwards School of Medicine, Huntington, USA; 3 Internal Medicine, Nawaz Sharif Medical College, University of Gujrat, Gujrat, PAK; 4 Internal Medicine/Pulmonology, Marshall University Joan C. Edwards School of Medicine, Huntington, USA

**Keywords:** bronchoscope, bronchitis

## Abstract

Tracheobronchopathia osteochondroplastica (TBPO) is a rare benign disease of unknown cause, in which multiple cartilaginous or bony submucosal nodules project into the trachea and proximal bronchi. It usually occurs in men in their fifth decade and can cause airway obstruction, bleeding and chronic cough; patients are more prone to post-obstructive pneumonia and chronic lung infection in some instances. We report a case of a 69-year-old female who presented with shortness of breath and lower extremity swelling over the past couple of weeks. Echocardiography (ECHO) was consistent with heart failure with preserved ejection fraction, and she was treated with diuretics accordingly. Imaging revealed persistent pleural effusions bilaterally, more pronounced on the right side. During the course of her hospitalization, the patient coded once and had to be resuscitated. She had bronchoscopy done and pathology was consistent with TBPO. In this condition, there are numerous osseous or cartilaginous submucosal nodules in the trachea and the main bronchus and nodules are formed due to the deposition of calcium phosphate that results in the proliferation of osseous and cartilaginous structures resulting in the obstruction of large airways. Treatment for the most part is supportive and resolves around bronchodilators for symptomatic relief.

## Introduction

Tracheobronchopathia osteochondroplastica (TBPO) is an idiopathic, rare, benign pulmonary condition with an incidence of 0.01 to 4.2 per 100,000 people [1]. It was first described in 1857 and observed in an autopsy of a patient with pulmonary tuberculosis. This condition is characterized by numerous osseous or cartilaginous submucosal nodules in the trachea and main bronchus. It usually occurs in men in their fifth decade and cause airway obstruction, bleeding and chronic cough, and patients are more prone to post-obstructive pneumonia and chronic lung infection in some instances. Nodules causing this condition are formed due to deposition of calcium phosphate [2]. Most patients are asymptomatic and are diagnosed incidentally. We present a case of TBPO diagnosed in a patient who presented to hospital with symptoms suggestive of acute decompensated heart failure.

## Case presentation

**Figure 1:**
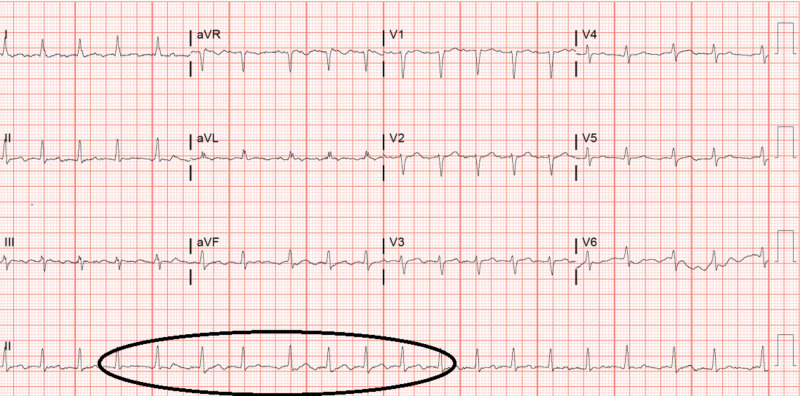
Electrocardiogram showing atrial fibrillation with rapid ventricular rate

We present a case of 69-year-old female with past medical history significant for hypertension, hyperlipidemia and diabetes who came to the hospital with shortness of breath and lower extremity swelling that had progressively worsened over the last two weeks. These symptoms were accompanied with weight gain, orthopnea and chest pressure. She denied any chills, fevers and cough. She denied any dizziness, wheeze or changes to her vision. She also denied any history of smoking in the past and did not have any history of any underlying cardiac or pulmonary disease. At home, she was taking allopurinol 100 mg daily for gout, glimepiride 4 mg oral tablet twice daily, nateglinide 120 mg and linagliptin 5 mg for diabetes, amlodipine 5 mg for hypertension and simvastatin 40 mg for hyperlipidemia. At the time of admission, her labs were unremarkable for any acute abnormality except for a B-type natriuretic peptide of 2,772. Electrocardiography showed atrial fibrillation with rapid ventricular response (Figure 1)

**Figure 2:**
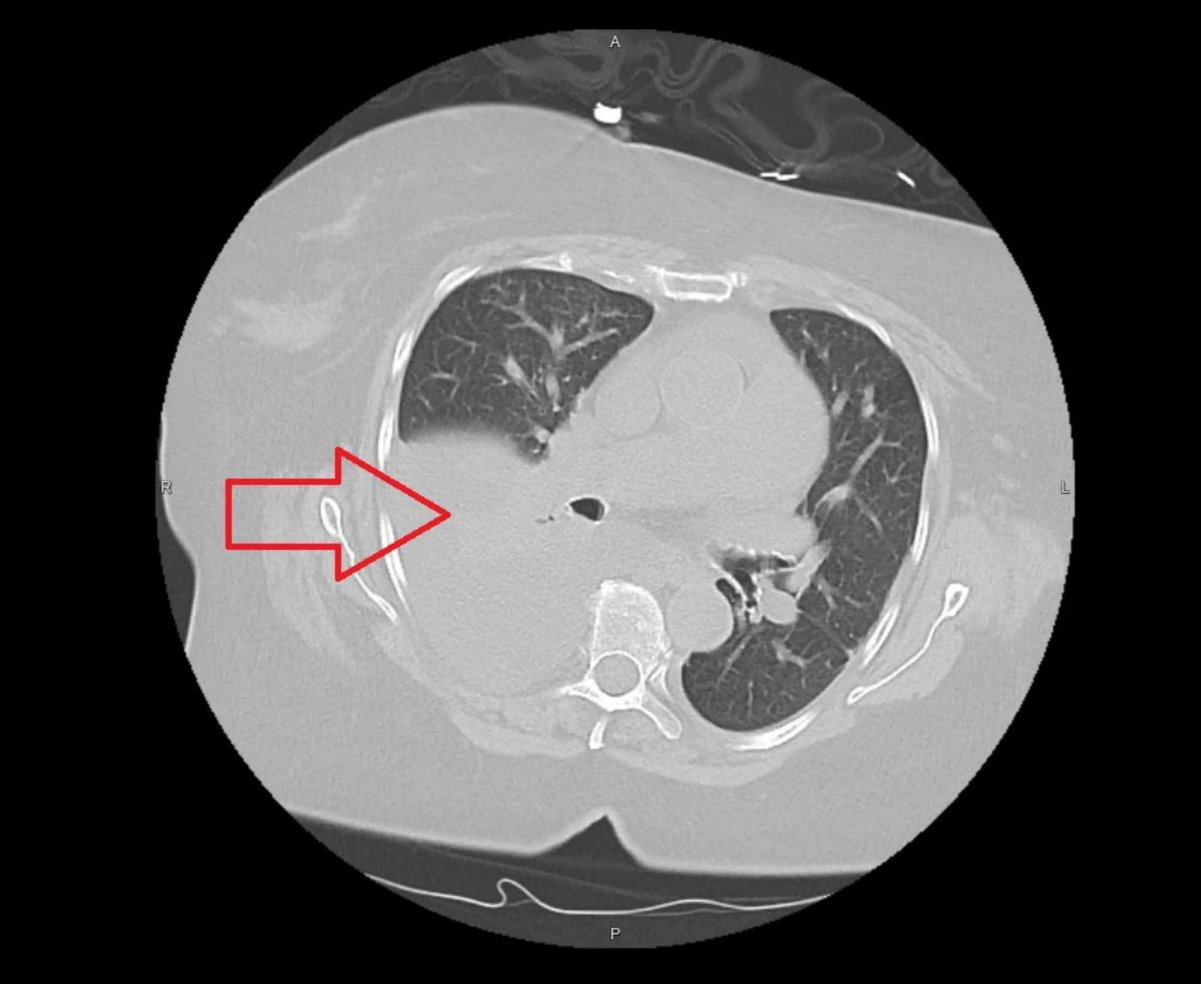
CT chest without contrast showing large right lung effusions (red arrow) with atelectasis and cardiomegaly

CT scan of chest without contrast showed pulmonary edema and pleural effusions right greater than left (Figure 2).

Cardiology was consulted and she was initially started on intravenous diuresis with 80 mg of furosemide twice daily which was later transitioned to bumetanide drip because of persistent volume overload. Echocardiography showed findings consistent with heart failure with preserved ejection fraction (EF). It also showed dilated left atrium with right ventricular systolic pressure of 52 mmHg and EF >55%. Pulmonology was consulted for persistent pleural effusion and thoracentesis was performed on day 4 of hospital admission. Approximately 1,100 mL of serous fluid was removed and fluid analysis revealed the effusion to be transudative in nature. Right heart catheterization (RHC) was performed and revealed a transpulmonary gradient of 24 mmHg which could not be explained by only left-sided disease; hence, a Swan-Ganz catheter was left in place for hemodynamic monitoring. Other findings from RHC revealed wedge pressures 28/20/23 mmHg, pulmonary artery pressure of 60/36/47 mmHg, right atrial pressure of 21/20/20 mmHg and right ventricular pressure of 57/12/14 mmHg; pulmonary vascular resistance of 3.3; cardiac output Fick's 9.7; cardiac index Fick's 4.06.

CT chest without contrast demonstrated large dependent pleural effusion on the right side and repeat thoracentesis was done. Approximately 1,000 mL of serous fluid was removed and pleural fluid analysis was unchanged from the one performed before. Post procedure film did not show any pneumothorax. The same day, the patient became bradycardic and then progressed to cardiac arrest with initial rhythm being asystole. She was resuscitated for about 8-10 minutes after which return of spontaneous circulation (ROSC) was achieved. A diagnostic and therapeutic bronchoscopy was performed. Repeat bronchoscopy was performed and revealed multiple small cysts and a large cast obstructing the right main stem consistent with plastic bronchitis that was removed. Post cardiac arrest, the patient required multiple pressor support, and repeat echo showed an EF of 20%-25%. Neurology was consulted because of her persistent areflexia and deemed her overall prognosis to be poor. Eventually, the family pursued comfort care measures for her and the patient passed away the following day.

## Discussion

TBPO is an idiopathic, rare, benign pulmonary condition with an incidence of 0.01 to 4.2 per 100,000 people [1]. It was first described in 1857 and was observed in an autopsy of a patient with pulmonary tuberculosis [2]. In TPBO, there are numerous osseous or cartilaginous submucosal nodules in the trachea and the main bronchus as implied by the name. The nodules are formed due to the deposition of calcium phosphate that results in the proliferation of osseous and cartilaginous structures resulting in the obstruction of large airways [3]. Most patients are asymptomatic and diagnosis is incidental based on bronchoscopy or during a troublesome intubation [4]. The mean age of diagnosis is 50 years, although it has been reported in a patient as young as nine years old [5].

TBPO is definitively diagnosed by bronchoscopy during which the endobronchial nodules are biopsied and sent for histopathological analysis. Patients who experience symptoms might undergo imaging in the form of a chest CT scan prior to an invasive procedure [6]. Bronchoscopy, however, is most definitive diagnostic tool. Imaging may show obstruction in the larger pulmonary airways. The severity of symptoms depends on the location and spectrum of obstruction of the airways. These diagnostic modalities may also be supplemented by spirometry and pulmonary function tests [6].

Leske et al. studied 41 subjects with chronic cough alongside dyspnea and sputum production to be the most common symptoms. Other symptoms include chronic cough, hemoptysis, wheezing, fever, weight loss, chest pain, dysphagia and dysphonia [7]. Treatment depends on disease presentation; asymptomatic cases are not treated, whereas symptomatic ones are treated appropriately. Conservative management modalities include air humidification, avoiding irritants of the respiratory pathway and infection control. Prior studies have indicated different invasive treatment modalities, yet the results have not been promising. A case with tracheal stenosis was treated with laser therapy with only moderate improvement, whereas another case which included the excision of nodules did not result in the improvement of atelectasis post-treatment. No specific therapy exists for this condition and management is usually directed towards relieving symptoms and treating pulmonary infections when indicated [8].

## Conclusions

TBPO is an idiopathic, rare, benign pulmonary condition with various presentations. Incidence is low and the disease is characterized by various manifestation. It is diagnosed with a bronchoscopy and treatment focuses on symptomatic relief. Our patient was incidentally found to have this condition where the overall clinical picture was clouded by acute decompensated heart failure complicated with asystole.
